# Spatial Distribution Characteristics and Risk Assessment of Soil Heavy Metals from Long-Term Mining Activities: A Case Study of the Fengfeng Mining Area

**DOI:** 10.3390/toxics13110969

**Published:** 2025-11-10

**Authors:** Le Ren, Wenyu Qi, Hongling Ye

**Affiliations:** 1 School of Life Science and Engineering, Handan University, Handan 056005, China; hdxyrl@hdc.edu.cn; 2 Shandong Key Laboratory of Water Pollution Control and Resource Reuse, School of Environmental Science and Engineering, Shandong University, Qingdao 266237, China

**Keywords:** long-term mining, HMs speciation, land degradation, ecological risk, spatial distribution

## Abstract

Long-term mining activities have introduced heavy metals (HMs) into the soil, ultimately threatening environmental sustainability. Precisely forecasting the spatial patterns of HMs and performing risk evaluations in mining regions are essential for efficient pollution control. In this study, 213 topsoil samples were collected from the Fengfeng Mining Area, which has a 150-year mining history. To determine the spatial distribution of soil HM speciation, correlation analysis was conducted by integrating landform types, and visualization was carried out through Kriging interpolation. Results indicate that the mean levels of Cd, Cu, Pb, and Zn exceed their respective background values by 6.48, 1.61, 4.79, and 4.35 times. The bioavailability sequence is Cd > Pb > Zn > Cu, with elevated levels of bioavailable Cd and Pb observed in the western hilly region. Based on the secondary phase to primary phase ratio (RSP) and the risk assessment code (RAC), Pb and Cd were identified as posing high ecological risks, whereas Cu and Zn do not cause severe contamination. This study provides a scientific foundation for industrial transformation and sustainable development in resource-exhausted cities.

## 1. Introduction

In coal mining districts, long-term and large-scale coal extraction has led to substantial environmental contamination, posing severe risks to the surrounding soil [1,2]. A substantial quantity of mine water and coal-based solid waste generated during coal mining can be easily leached into the soil by rainfall, eventually accumulating and degrading the soil environment [3]. Coal-based waste (such as coal gangue, fly ash, furnace bottom slag, and desulfurization gypsum) has an annual new emission of 797 million tons in China and generally contains heavy metals (HMs) [4]. The current disposal of coal-based waste is mainly managed through landfilling and stockpiling, leading to the leaching of hazardous substances into soil and aquatic environments [5]. In addition, stacking, grinding, blasting, and crushing coal ore could accelerate the weathering of coal-derived waste materials, ultimately leading to acidic mine drainage characterized by a low pH [6]. Conversely, the leakage of acidic mine drainage exacerbates the inflow and the leaching of HMs in the soil [7,8]. Therefore, in mining areas with long-term operational activities, toxic, persistent, and bioaccumulative HMs (e.g., cadmium, lead, copper, zinc, etc.) inevitably emerge as the primary contaminants requiring continuous monitoring.

Soil plays a crucial role in regulating the mobility and bioavailability of HMs, thereby influencing their transport to water, animals, and plants [9]. Hence, assessing the degree of soil HM pollution can provide valuable information for regional planning and sustainable development during the transformation of coal mining areas. Deng et al. [10] revealed that abandoned industrial sites exhibit significant spatial heterogeneity in HM pollution, presenting distinct horizontal and vertical aggregation patterns. Adnan et al. [11] found that smelting generates high concentrations of HMs in slag and dust, resulting in heterogeneous spatial distribution of HMs at an abandoned smelting site. In addition, Zhu et al. [12] observed HM enrichment in surface soil and the Carboniferous–Permian coal seams in the Guobei coal mine. While Qureshi et al. [13] suggested that coal mining activities enhance the toxic metals contamination in gangue soil reclamation. These findings suggest potential concerns regarding soil HM pollution caused by long-term coal mining due to their ore veins, production, processing, and stacking and treatment of coal-based solid waste. Nevertheless, there are relatively few studies on the distribution of HMs in the soil of long-term operating coal mines. Meanwhile, HMs in soil can exist as free ions or complexes bound to soil minerals, organic matter, or microorganisms, or within the crystal lattice of soil minerals, and these forms may transform within the soil environment [14]. Notably, the biotoxicity of HMs is strongly influenced by their speciation, which determines their environmental behavior and directly affects their mobility and toxicity [15]. Although certain HM forms in soil may be relatively stable, they may still be transformed into relatively active components under specific geochemical conditions. Consequently, spatial heterogeneity in soil HM distribution can occur in areas with complex topography and human activities, driven by variations in multiple influencing factors, such as HM migration behaviors.

China possesses abundant coal resources and ranks third globally in terms of proven reserves. In traditional coal mining regions, industrial transformation is essential for sustainable development due to coal resource depletion, making soil ecological protection and remediation critically important. Prolonged resource extraction and the associated industrial development have impacted HM speciation differently across various landforms [16,17]. However, there are limited studies on soil HM pollution in coal mining areas with long-term mining activities [18]. The Fengfeng Mining Area, located in the south of Hebei Province, is a major coal production base in China. Long-term coal mining (150 years) has led to coal-based solid waste accumulation and continuous acidic mine drainage leakage, threatening the surrounding soil’s ecosystem. Previous studies on the long-term environmental and ecological impacts of mining in this region mainly focused on groundwater contamination [19,20], lacking systematic research on HM distribution and risk assessment in regional soils. Meanwhile, the depletion of coal resources has prompted regional urban transformation. Therefore, comprehensive investigations into the contamination levels, spatial patterns, and potential ecological risks of HM chemical speciation across different landforms in the whole mining area are urgently needed to support ecological restoration and sustainable land-use planning.

The objectives of this study are as follows: (1) to investigate the spatial heterogeneity characteristics of HMs in soil; (2) to utilize statistical analysis and geographic information systems for quantitative analysis and visualization; (3) to assess the HMs’ ecological risks using the secondary phase to primary phase ratio (RSP) and the risk assessment code (RAC).

## 2. Materials and Methods

### 2.1. Study Area

**Figure 1:**
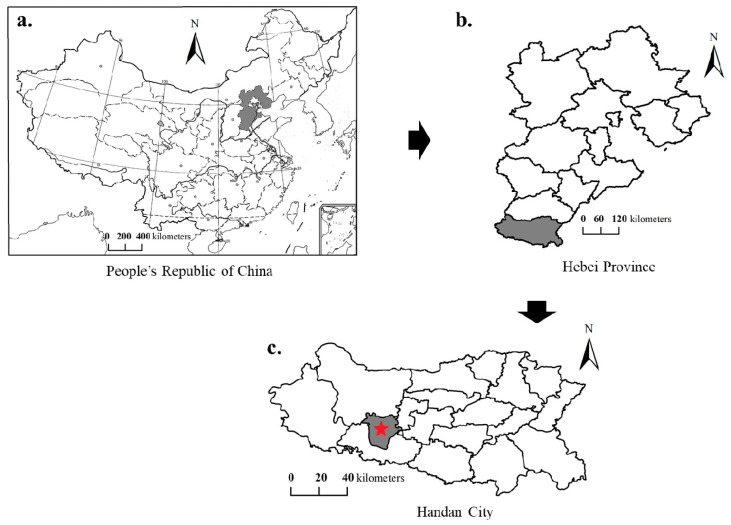
Map of the Fengfeng Mining Area, China. (**a**) Map of the People’s Republic of China; (**b**) Map of Hebei Province; (**c**) Map of Handan City.

**Figure 2:**
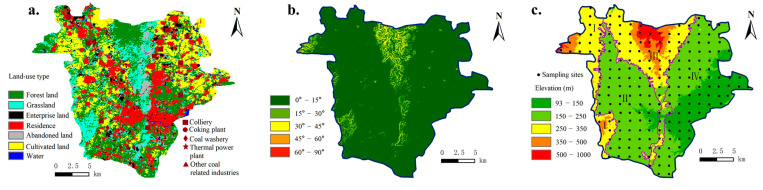
The land-use type (**a**), landform (**b**) and sampling sites distribution (**c**) of Fengfeng Mining Area.

Fengfeng Mining Area is located in southern Hebei Province in North China, under the jurisdiction of Handan City (Figure 1). It is a typical resource-based industrial and mining region characterized by the distribution and exploitation of mineral resources. It is a major coal-producing region, it supplies millions of tons of coal annually to meet national energy demands. Centered on coal extraction, its core industries primarily include coal mining and coal-related chemical processing, such as coal washing, coking, and power generation. (Figure 2a) The permanent resident population totals 406,100 predominantly distributed along both sides of the central mountain range within the region. Situated at the transitional zone between the Taihang Mountains and the North China Plain, the area features a warm, semi-humid to semi-arid continental monsoon climate, with an average annual temperature of 14.1 °C and mean annual precipitation of approximately 627 mm. The district spans a total area of 353 km^2^. Within the study area, there are 17 coal mine hills, with a combined storage capacity of approximately 36.6 million tons and covering an area of roughly 1.05km^2^.

According to the landform type, the study area is divided into four subareas (Figure 2b): I—Western hilly area, with a mean slope of about 30° and an average elevation of 313.35 m, mainly including cultivated land and grassland; II—Central basin area, with a slope of 0–20° and an average elevation of 207.31 m, where there are many industrial and mining enterprises in the north, with a large amount of coal gangue, and which is an ecological restoration area; III—Central mountainous area, with a slope of more than 30° and a mean elevation of 333.62 m, mainly including forest land and grassland; IV—Eastern inclined plain area, with a slope of less than 15° and a mean elevation of 178.32 m. This is the main farming area in the study area. There are several large coal mines in the north, and Dongwushi reservoir in the south. The land in Fengfeng Mining Area was divided into V-shaped areas. The most extensive area is the basic farmland protection area (Figure 2b).

### 2.2. Soil Sampling and Measurement

The grid sampling method was used for determining soil sampling points in this study. The spacing of the soil sampling grid was 1.5 km, and a total of 213 sampling points were determined (Figure 2c). The soil samples were collected from the topsoil (0–20 cm). At each sampling point (5 m × 5 m), samples were taken at the four vertices and centers, and then mixed thoroughly to a composite sample (~2 kg). After the samples were transported into the laboratory, they were air-dried and ground to pass a 0.15 mm sieve for subsequent analysis. For specific information on the sampling points, please refer to the appendix. The relevant altitude and slope data of the soil sampling points are downloaded from the “Geospatial Data Cloud” (http://www.gscloud.cn/). All data are at a resolution of 30 m.

The speciation of HMs (Cd, Pb, Cu, and Zn) was determined with the Tessier five-step extraction method [21] to extract exchangeable (EX), carbonate-bound (CA), Fe-Mn oxide-bound (OX), organic-matter bound (OR), and residual (RE) speciation, respectively. In brief, exchangeable HMs were extracted with 1.0 M MgCl_2_; carbonate-bound HMs were extracted with 1.0 M NaAc (pH 5.0); and HMs bound to iron-manganese oxides were extracted with 0.04 M NH_2_OH·HCl. The organic matter-bound HMs were extracted with 3 mL of 0.02 M HNO_3_ and 5 mL of 30% H_2_O_2_ (pH 2.0, 85 ± 2 °C). The residue of the previous step was digested with a mixture of HNO_3_: HCl: HF (6:3:2). The above metal speciation in the digestive solution were measured using an atomic absorption spectrophotometer (AA240FS, Agilent, Santa Clara, CA, USA). For repeatability and accuracy, three replicates were set for each sample and the standard soil samples (NSA-2, Institute of geophysical and geochemical exploration, Chinese Academy of Geological Sciences) were used for quality control in each step.

### 2.3. Ecological Risk Assessment

Methods such as the pollution index (PI), geo-accumulation index (Igeo), and risk index (RI) are commonly employed to assess the environmental risks associated with total heavy metal concentrations in individual soil samples. To enable a more systematic evaluation of the environmental risks posed by different HM speciation, this study incorporates the RSP and RAC methods.

The RSP method is widely utilized in the ecological risk assessment based on the sequential extraction [22,23]. The calculation formula is as follows:

$$RSP=\frac{\gamma_{\sec}}{\gamma_{\mathrm{prim}}}$$

where *RSP* is the ratio of secondary phase to primary phase, representing the ecological risk of HMs; *γ*_prim_ is the mass concentration of HMs in the residual form present in the primary mineral lattice, mg·kg^−1^. *γ*_sec_ is the mass concentration of HMs in the soil secondary phase (transformed through a variety of physical and chemical processes after being released from primary mineral), mg·kg^−1^. It includes weak acid soluble (EX + CA), reducible (OX), and oxidizable (OR) HMs. The soil ecological risk is divided into four levels according to the RSP value: RSP ≤ 1, non-pollution; 1 < RSP ≤ 2, slight pollution; 2 < RSP ≤ 3, moderate pollution; RSP > 3, severe pollution. The maps in the article were all generated using the ordinary Kriging interpolation method, with ArcGIS 10.2 (ESRI, Redlands, CA, USA).

The RAC method places greater emphasis on the weak acid soluble speciation and evaluates its environmental risk by calculating the ratio of this speciation to the total concentration, using the following formula:

$$RAC=\frac{\gamma_{F1}}{\gamma_{T}}\times 100\%$$

where *RAC* is the mass speciation of weak acid soluble content in the total amount, %; *γ_F_*_1_ is the content of weak acid soluble HMs, mg·kg^−1^; *γ_T_* is the sum of all speciation of HMs in soil, mg·kg^−1^. Based on the RAC value, the risk level is categorized into five tiers: RAC < 1% indicates no risk; 1% ≤ RAC < 10% represents low risk; 10% ≤ RAC < 30% corresponds to medium risk; 30% ≤ RAC < 50% signifies high risk; and RAC ≥ 50% denotes extremely high risk.

### 2.4. Statistical Analyzes

The differences between groups were conducted using a one-way ANOVA or Student’s *t*-test followed by Tukey’s test in SPSS software (version 27.0, IBM, Armonk, NY, USA). The significance levels of *p*-values < 0.05 were employed to determine statistical significance or extreme significance for all comparisons. The confidence interval was set at 95% in thestatistical analysis. 

## 3. Results and Discussion

### 3.1. Spatial Distribution Patterns of HMs in Soil

The spatial distribution characteristics of HMs, including Cd, Cu, Pb, and Zn, in the study area were analyzed using descriptive statistics (Table 1). Compared with the soil background values for Hebei Province [24], the average concentrations of Cd, Cu, Pb, and Zn were approximately 6.48, 1.61, 4.79, and 4.35 times higher, respectively. These elevated levels also exceed the national soil background values reported by Wang et al. [25], indicating significant accumulation of these HMs in the soils of the Fengfeng Mining Area and underscoring the need to prioritize Cd, Cu, Pb, and Zn as key pollutants for targeted control. Additionally, the coefficient of variation (CV) analysis was performed to assess the spatial variability of HMs to distinguish between anthropogenic and natural sources [26,27]. The CV values for Pb (29.02%) and Zn (25.56%) suggest moderate variability, whereas higher CVs for Cd (56.41%) and Cu (44.66%) indicate pronounced fluctuations, likely attributable to anthropogenic activities.

**Figure 3:**
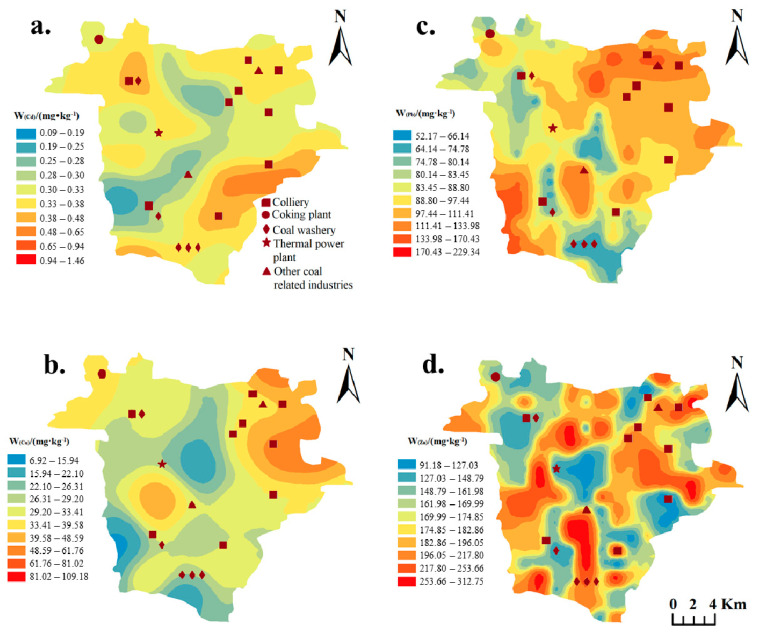
Spatial distribution of the total concentration of four heavy metals. (**a**) Cadmium, (**b**) copper, (**c**) lead, (**d**) zinc.

**Figure 4:**
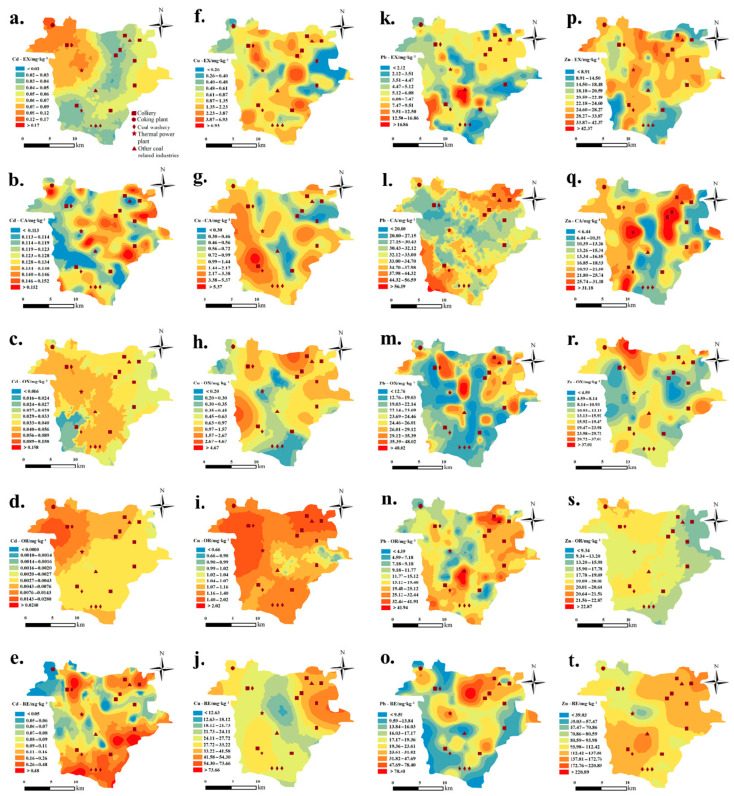
Spatial distribution heterogeneity of heavy metals across different soil components in the Fengfeng Mining Area. Cd speciations, (**a**–**e**); Cu speciations, (**f**–**j**); Pb speciations, (**k**–**o**); Zn speciations, (**p**–**t**). EX, extract exchangeable; CA, carbonate bound; OX, Fe-Mn oxide bound; OR, organic matter-bound; RE, residual speciation.

To further analyze the spatial distributions of various speciation of HMs, GIS mapping techniques were used based on the ordinary Kriging interpolation method. In brief, the concentrations of these four HMs exhibit significant spatial heterogeneity across the study area (Figure 3 and Figure 4). Cd in the soils of Fengfeng Mining Area has a total concentration ranging from 0.10 to 1.46 mg/kg with a mean of 0.39 mg·kg^−1^. The coal in China contains approximately 0.43 mg·kg^−1^ [28]. During coal combustion, Cd readily undergoes phase transformation from solid to gas due to its semi-volatile nature. As the flue gas cools, cadmium condenses onto the surfaces of aerosols and ash particles, subsequently depositing into the soil via atmospheric pathways [29]. The available forms in soil, Cd-EX, and Cd-OR were relatively high in the northwest of the Western hilly area (Figure 4a,d). Meanwhile, there are many local coal-based industries (such as coking plants, coal washeries and power plants). Acidic mine drainage can promote the transformation of cadmium into a more leachable form. The concentration of Cd-EX was low (below 0.04 mg·kg^−1^) in the central mountainous and southwest hilly areas, relatively high in the Eastern inclined plain and the southern part of the study area, and relatively low (below 0.07 mg·kg^−1^) in the Western hilly area. The difference in Cd-OX concentration among landform types was small, and the concentration was only low (below 0.03 mg·kg^−1^) in the southern part of the Western hilly area (Figure 4c). Meanwhile, the probability of soil Cu exceeding the Hebei Province’s standard in this area is 86.92%, which is the lowest among the four HMs (Table 1). Similarly, Liu et al. [30] indicated that the pollution level of Cd was the highest in the park soil and dust of a coal resource-based city, while that of Cu was relatively low. As an essential trace element for various physiological and biochemical processes of plants, Cu is more easily absorbed and transported by plants than Cd and Pb, which might be the reason for the lower soil Cu contamination [31]. The concentration of Cu-EX was higher in the Central mountainous area and the northern part of the Central basin (Figure 4f). Cu-CA was predominantly found in the Western hilly area (Figure 4g). Cu-OX was mainly distributed in the Western hilly area and the northern part of the Central mountainous area, while Cu-RE was concentrated in the northeast of the study area (Figure 4h,j).

Regarded as one of the least mobile HMs in soil, Pb can be accumulated through a range of natural processes [32]. In the Fengfeng Mining Area, it may be released from mine tailings and subsequently interact with surface and groundwater, leading to elevated concentrations of Pb in the soil. Notably, the weighted average Pb content in Chinese coal is calculated to be 14.03 mg·kg^−1^ [33], which resulted in the coal industry releasing significant amounts of lead into the atmosphere, with soil lead contamination being especially pronounced in the Fengfeng Mining Area. The average soil lead content in the Fengfeng area is 2.4 times higher than the average soil lead concentration in Chinese coal mines reported by Liu et al. [34]. The spatial distribution of different speciation of Pb in the study area showed strong regularity (Figure 3 and Figure 4k–o). Except for the residual speciation, the contents of Pb speciation were generally higher in the north of the Eastern inclined plain and the south of the Western hilly area. The factors leading to the distribution centers of different speciation of lead in these two places were quite different. In the north of the Eastern inclined plain, there were many still-exploited coals mines, such as Dashe Mine, Xuecun Mine, Shucun Mine, and Xiaotun Mine. These mines accumulated many gangues and increased the content of secondary phase Pb in the soil. Pb-RE was mainly distributed along the Central mountainous area. Especially, the distribution center of Pb-RE in the northern part of the central mountainous area, which is rarely visited by people, was less affected by human activities.

As a major component in the atmospheric particulates, Zn could readily accumulate in soil through mining-related dust deposition [35]. From the spatial distribution map of different speciation of Zn (Figure 3 and Figure 4p–t), Zn-EX was distributed only in parts of the Central basin area, the Central mountainous area, and the Eastern inclined plain area, while its concentration was lower in the southern part of Western hilly area, and the southern end and northern part of the Eastern inclined plain. Belmer and Wright [36] found that the mean Zn concentration rose from 8.6 µg/L upstream to 83.4 µg/L downstream of the mine waste discharge points. At the same time, the discharge and leakage of acidic wastewater have also affected the transformations of Zn speciation [37]. Therefore, the area with more coal mining enterprises also had higher Zn-EX concentrations. The Zn-CA content was higher on both sides of the Central mountainous area and in the Western hilly area. Zn-OX was concentrated on the north side of the Central mountainous area. The concentration of Zn-OR was low only at the northeast edge of the Eastern inclined plain, and there was little difference in other areas. Zn-RE was mainly distributed in the north of the Eastern inclined plain area, the Central basin area, and the south of the Western hilly area.

### 3.2. Distribution Characteristics of HM Speciation Based on Landform

**Figure 5:**
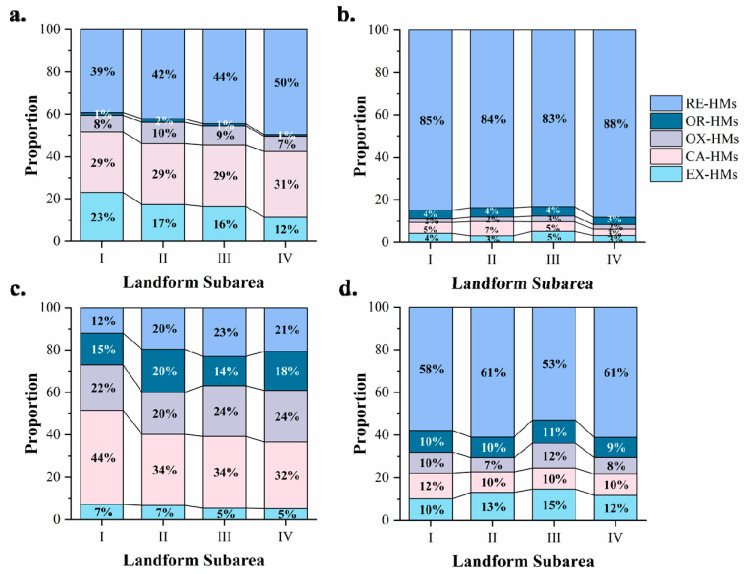
Spatial distribution heterogeneity of heavy metals across different soil speciation in the Fengfeng Mining Area. (**a**) Cadmium, (**b**) copper, (**c**) lead, (**d**) zinc. I—Western hilly area; II—Central basin area; III—Central mountainous area; IV—Eastern inclined plain area. EX, extract exchangeable; CA, carbonate-bound; OX, Fe-Mn oxide-bound; OR, organic matter-bound; RE, residual speciation.

At the regional scale, landforms that are closely associated with geological structures, mineral concentration belts, and specific underlying strata serve as valuable indicators for assessing the spatial heterogeneity of soil HMs [38]. To further analyze the distribution characteristics of different HM speciation, we partitioned the research area into four subareas according to the topography and performed one-way ANOVA for each speciation (Table S1). Subarea Ⅰ exhibits the highest exchangeable levels of Cd and Pb, which are readily leached and can be accumulated through biological utilization. In Subarea Ⅱ, high contents of Zn-EX, Cu-CA, and Pb-RE are observed. Subarea Ⅲ also shows relatively high levels of Zn-EX and Zn-OX. As for Subarea Ⅳ, it has high contents of Cd-CA, Pb-RE, and Cu-RE. From the proportion of each speciation (Figure 5), Cd, Cu, and Zn were predominantly in the residual speciation. Specifically, the residual speciation of Cu and Zn (Cu-RE and Zn-RE) accounted for over 50% of all four landform types. The proportion of the residual speciation of Cd (Cd-RE) ranged from 36.19% to 40.5%, slightly higher than that of the Fe-Mn oxide-bound speciation (Cd-OX). Residual speciation of HMs, mainly influenced by mineral composition, rock weathering, and soil erosion, is typically not subject to leaching due to soil moisture content, pH levels, or biological activity [11]. Among the four HMs analyzed, Pb had the lowest proportion in the residual speciation in the study area. The highest proportion of the residual speciation of Pb was 21.82% in the Central mountainous area, and it was lower in other landforms. Pb was mainly in the carbonate-bound speciation, with the highest proportion of 43.89% in the Western hilly area and the lowest of 32.37% in the Eastern inclined plain area.

The Fe-Mn oxide-bound and organic-matter bound (OX and OR) HMs are potential bioavailable speciation which could be released and utilized by organisms when in a reducing or oxidizing environment [39]. In this study, the OX and OR forms of Pb accounted for the largest proportion among the four HMs. The proportions of Pb-OX and Pb-OR in each of the four subareas were more than 19.66% and 13%, respectively. These results are mainly because Pb is easily adsorbed on goethite to form a stable complex and thus is not easy to release under oxidation conditions, which is consistent with other studies [40]. The concentration of Pb-OX in the Eastern inclined plain was significantly higher than that in the Central basin area (*p* < 0.05). Meanwhile, the concentration of Pb-OR in the Central basin and the Eastern inclined plain area was significantly higher than that in the Central mountainous area, which might be related to the high clay content in these areas. Pb was found to be strongly adsorbed by the clay minerals in soil, to such an extent that the mobility of Pb was not enhanced with decreasing pH values [41]. Therefore, Pb was an important potential pollution element in the region. The other three elements occupied a low proportion in the reducible and oxidizable speciation. Especially, the reducible speciation of Cu accounted for less than 3% in the four landforms, indicating that Cu had poor reducibility and posed little potential harm to the study area.

Exchangeable speciation and carbonate-bound (EX and CA) speciation, also known as the weak acid soluble speciation, could release HMs in soil into a directly bioavailable and highly harmful state when environmental conditions (e.g., pH) change [42]. Notably, the weak acid soluble state of Cd and Pb accounts for a relatively large proportion among the total content, which exceeds 43% and 37%, respectively. The proportion of the weak acid soluble speciation of Cd and Pb was the highest in the Western hilly area (Cd—53.54% and Pb—50.50%) and the lowest in the Eastern inclined plain area (Cd—49.86% and Pb—37.57%). The high content of weakly acid soluble Cd may be due to the formation of cadmium carbonate complexes [43]. In the study area, the soil exhibited a moderately alkaline condition overall due to the relatively high limestone content in its parent material, which partially limited the availability of HMs. However, with the extensive development of mineral resources, particularly the long-term accumulation of coal gangue rich in iron (Fe) and sulfur (S), these materials may undergo environmental chemical reactions and produce acidic mine drainage, thereby gradually affecting the bioavailability of HMs [7]. Shylla et al. reported that acidic tailings and mine drainage leaching cause acidification of the water near the mine in northeast India [44]. Another study on the evaluation of surface water quality following mine closure in a coal mining region indicates that HM concentrations in most surface water samples exceeded regulatory standards, with pollution levels being more severe near the mines and gradually decreasing with distance from the source [45].

### 3.3. Risk Assessment Based on Soil HM Speciation

The ecological risk of HMs (Cd, Pb, Cu, and Zn) in the soil of four subareas was evaluated by the RSP and RAC methods (Table 2). The RSP method focuses on the ratio of HMs content in the secondary phase relative to that in the primary phase, highlighting pollution risks associated with both directly bioavailable and potentially bioavailable speciation [46,47]. Severe pollution frequencies across all sites are as follows: Pb (69.95%) > Cd (26.76%) > Zn (2.35%) > Cu (0.00%) (Figure S1). The average values of Cd and Pb across all sampling sites in the Fengfeng Mining Area exceeded 3 (reaching 7.50 and 7.17, respectively), indicating severe pollution in soils with high potential ecological risk (Table 2 and Figure S2). In contrast, the average values of Cu and Zn were both below 1 (0.22 and 0.90, respectively), suggesting that these metals do not meet the pollution thresholds defined by the RSP method. The maximum RSP values of Cd and Pb in the Western hilly area were significantly higher than those in the other three landform types. This subarea features a large expanse of grassland, a thin soil layer, poor water-holding capacity, frequent dry–wet alternations, and intense weathering. Long-term mining activities in this environment have intensified the accumulation of secondary phase Cd and Pb in the soil. Meanwhile, the RSP values of Cu in the Western hilly area and the Central mountainous area were significantly higher than those in the Eastern inclined plain area, where no pollution risk was detected. This further suggested that Cu is relatively inert in the soil environment and less affected by soil conditions.

In contrast to the RSP method, the RAC method emphasizes the proportion of weak acid-extractable HMs relative to the total concentration. Pollution assessment based on the bioavailability-derived RAC method revealed that Cd, Cu, Pb, and Zn maintained contaminated status across all subareas (Table 2). Contamination frequencies with of high and extreme risks ranked among all sites as follows: Pb (85.92%) > Cd (85.45%) > Zn (17.84%) > Cu (4.69%) (Figure S3). Notably, all soil samples collected from the sampling points exhibit a moderate or higher risk of leaching for more than two HMs. Overall, the pollution caused by Cd and Pb was the most severe, which suggested that their ecological risk was relatively serious and needed attention (Figure S4). It may be because Cd and Pb were relatively active, leading to the proneness of Cd^2+^, Pb^2+^, and Ca^2+^ to substitute with each other during the carbonate formation process, which in turn results in a high content of the weak acid-extractable speciation in the soil [48]. The weak acid-extractable speciation of Cd posed a high or extremely high risk in the four landform types. The proportion of the weak acid-extractable speciation of Pb in the Western hilly area was significantly higher than that in the other three landform types, presenting an extremely high risk in the Western hilly area and a high risk in the other three landform types, whereas Cu and Zn posed medium and low risks, respectively, in the four landform types.

### 3.4. Spatial Risk Assessment Based on the Kriging Model

**Figure 6:**
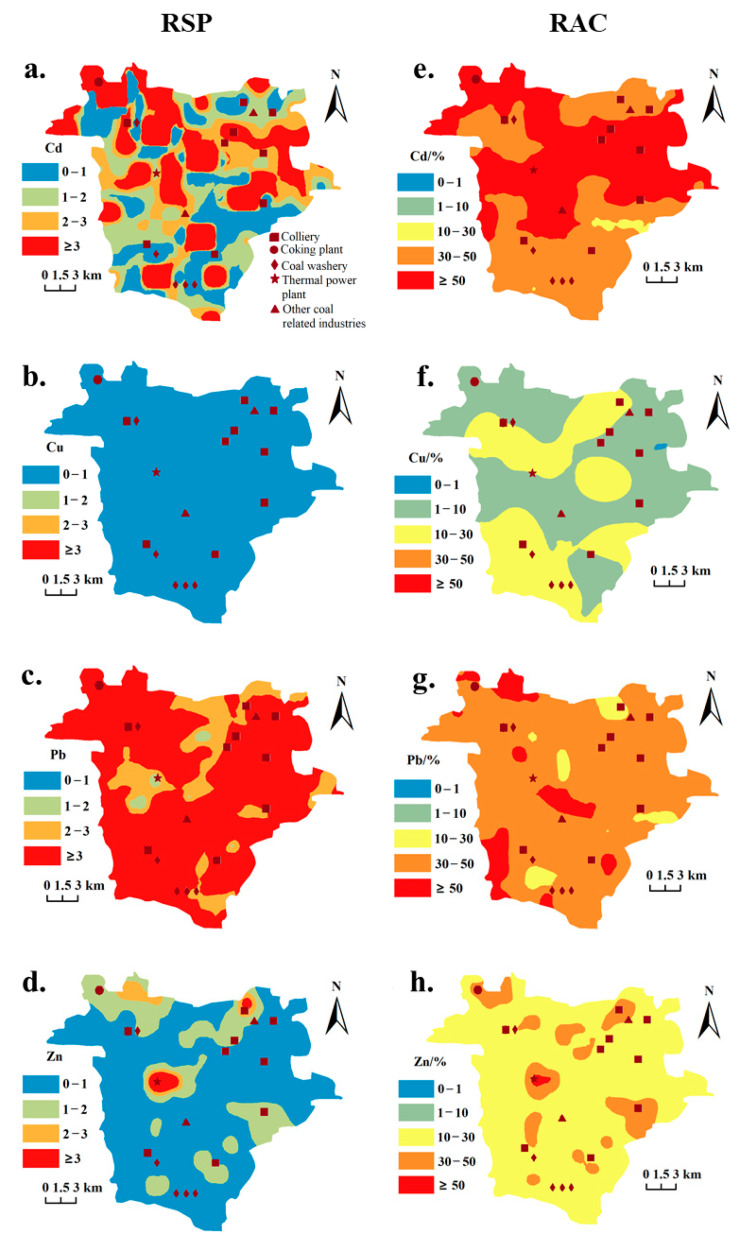
Spatial distribution of environmental assessment based on risk coding method (RSP, **a**–**d**) and the ratio of secondary phase and primary phase (RAC, **e**–**h**) in the study area.

The spatial distribution of risk assessments for Cd, Cu, Pb, and Zn for the topsoil was evaluated by RSP and RAC methods based on the Kriging model (Figure 6). Based on the calculation and spatial prediction results of the RSP method, the moderately and highly polluted areas of Cd were distributed in a typical patchy pattern, which was highly consistent with the distribution of major industrial and mining enterprises. Except for some areas in the middle and north of the Central mountainous area and part of the Central basin area, the remaining areas for Pb were heavily polluted. Cu exhibited a non-polluted status across the entire study area, whereas Zn showed heavy pollution in the middle of the Central basin area, possibly due to the large amount of alkaline fly ash released by local thermal power plants. There were some slightly polluted areas at the junction of the Central mountainous area and the Western hilly area and in the north of the Eastern inclined plain Area. The results calculated by the RAC method indicate that high and extremely high risks caused by Cd and Pb were almost ubiquitous in the study area. Only the risk level of Pb in the Western hilly area was significantly higher than that in other landforms (*p* < 0.05), while there were no significant differences in the other three HMs among different landform types. Therefore, it can be inferred that the spatial distribution of local weak acid soluble HMs was more likely attributable to geochemical factors and less influenced by human factors. For Cu, most of the study region was at medium and low risks, with no high-risk areas. High and very high risks for Zn were mainly scattered in the Central basin area, Central mountainous area, and the Eastern inclined plain, and medium risks prevailed in most of the region. Moreover, there was no obvious spatial pattern among different landform types in the study area.

### 3.5. Perspectives and Limitations

Soils, due to their higher clay content which enhances the adsorption of HMs, generally exhibit a greater potential ecological risk compared to rocks and coal gangue [49]. Copper and zinc can be toxic to plants at elevated concentrations in soil, whereas cadmium and lead (non-essential metals) pose toxicity risks to living organisms even at low levels [50]. The Fengfeng Mining Area is characterized by a high degree of overlap among population density, coal mining activities, and soil HM contamination. Inadequate agricultural planning in this region may result in the uptake of HMs by crops, thereby posing serious threats to human health. In addition, Yu et al. found elevated concentrations of HMs in subsidence ponds within the Huaibei coal mining area, posing significant health risks associated with consuming crucian carp harvested from these water bodies [51]. The Dongwushi Reservoir in the study area serves as a critical water supply source for both industrial and agricultural activities in Handan City. Therefore, the elevated HM levels in aquatic environments resulting from soil contamination in mining areas cannot be overlooked.

Currently, Fengfeng Mining Area is primarily undergoing industrial transformation towards characteristic planting and tourism. The results of this study indicate that the Western hilly area has a relatively high risk of Cd and Pb pollution. It is essential to control and remediate bioavailable HMs in the soil and implement vegetation restoration to prevent these two HMs from entering the biosphere and posing risks to human health. For the spot-like HM pollution in the soil of other areas, targeted remediation can be adopted. Moreover, in regional planning, considering the pollution status of HM forms in the soil, priority should be given to the prevention and control of HM pollution in the Western and Central mountainous areas, which can be designated as ecological restoration zones.

Continuous mining activities in the Fengfeng area over the past 150 years have led to the unavailability of comprehensive data on the types and quantities of extracted minerals. Consequently, a limitation of this study lies in the inability to quantitatively analyze the influence of different mineral types on the distribution of soil HMs. In the future, additional studies will be needed to focus on alleviating soil HM pollution and supporting urban transformation planning in the Fengfeng Mining Area.

## 4. Conclusions

This study investigated the spatial distribution of soil HM (Cd, Cu, Pb, and Zn) speciation and ecological risk indexes. The proportion of bioavailable HMs from high to low was Cd > Pb > Zn > Cu. The bioavailable speciation contents of Cd and Pb in the Western hilly area were significantly higher than those in the Eastern inclined plain area, while the bioavailable speciation of Zn in the Western hilly area was significantly lower than that in other landforms. Based on the RSP and RAC methods, Cd and Pb exhibit relatively high ecological risks and pollution levels, making them the primary HM contaminants in the soils in the Fengfeng Mining Area. Notably, Pb poses significant direct and potential hazards. The pollution risks associated with Cd and Pb in the Western hilly region are markedly higher than those observed in the other three subareas. Specifically, the concentrations of Cd-EX and Cd-OR are particularly elevated in the northern part of the Western hilly area. Meanwhile, Cu and Zn have not yet resulted in severe pollution in the region.

## Figures and Tables

**Table 1:** Descriptive statistics of heavy metal contents of soils in Fengfeng Mining Area.

Heavy Metals in Soil Layer	Cd (0–20 cm)	Cu (0–20 cm)	Pb (0–20 cm)	Zn (0–20 cm)
Minimum content (mg/kg)	0.10	7.03	52.17	91.18
Maximum content (mg/kg)	1.46	113.87	229.33	312.75
Mean content (mg/kg)	0.39	33.79	98.23	183.26
Standard deviation	0.224	15.09	28.51	46.84
Coefficient of variation (%)	56.41	44.66	29.02	25.56
Background values of soil in China (mg/kg)	0.074	20.0	23.6	67.7
Percentage of sampling points exceeding the background values (%)	100	86.45	100	100
Background values of soil in Hebei Province (mg/kg)	0.06	21.00	20.50	71.90
Percentage of sampling points exceeding the background values (%)	100	86.92	100	100
Technical requirements of planting soil for greening	0.8	300	300	350
Percentage of sampling points exceeding the technical requirements (%)	0.00	0.00	0.00	0.00

**Table 2:** Ecological risk assessment based on heavy metal speciation in soil.

**Method**	**Landform Type**	**Cd**		**Cu**	
RSP	I	33.93 ± 124.28 a	HP (9-8-2-7)	0.26 ± 0.18 a	NP (26-0-0-0)
	II	2.74 ± 3.60 b	MP (15-16-12-15)	0.23 ± 0.17 ab	NP (57-1-0-0)
	III	6.87 ± 14.86 b	HP (12-12-6-17)	0.27 ± 0.22 a	NP (47-0-0-0)
	IV	2.84 ± 5.33 b	MP (24-20-20-18)	0.18 ± 0.16 b	NP (82-0-0-0)
	Total	7.50 ± 44.54	HP (60-56-40-57)	0.22 ± 0.18	NP (212-1-0-0)
RAC	I	53.54 ± 18.45% a	EHR (0-0-0-13-13)	11.16 ± 7.81% a	MR (0-13-12-1-0)
	II	50.44 ± 16.33% a	EHR (0-0-5-21-32)	10.52 ± 7.15% a	MR (2-31-22-3-0)
	III	52.72 ± 22.22% a	EHR (0-1-9-11-26)	10.80 ± 9.16% a	MR (0-30-14-3-0)
	IV	49.86 ± 19.30% a	HR (0-1-15-16-50)	7.76 ± 8.33% a	LR (8-56-15-3-0)
	Total	51.10 ± 19.06%	EHR (0-2-29-61-121)	9.59 ± 8.24%	LR (10-130-63-10-0)
**Method**	**Landform Type**	**Pb**		**Zn**	
RSP	I	20.57 ± 32.94 a	HP (0-0-1-25)	0.79 ± 0.31 a	NP (21-5-0-0)
	II	5.99 ± 6.35 b	HP (1-2-15-40)	0.93 ± 1.15 a	NP (48-6-2-2)
	III	5.48 ± 5.94 b	HP (1-0-18-28)	1.10 ± 0.65 a	SP (29-12-5-1)
	IV	4.72 ± 3.17 b	HP (0-6-20-56)	0.81 ± 0.62 a	NP (67-11-2-2)
	Total	7.17 ± 13.25	HP (2-8-54-149)	0.90 ± 0.49 a	NP (165-34-9-5)
RAC	I	50.51 ± 7.81% a	EHR (0-0-0-12-14)	22.60 ± 7.24% a	MR (0-0-24-2-0)
	II	40.77 ± 10.97% b	HR (0-1-6-41-10)	24.39 ± 9.66% a	MR (0-0-49-7-2)
	III	41.04 ± 14.02% b	HR (0-0-12-23-12)	25.29 ± 7.83% a	MR (0-0-33-14-0)
	IV	37.57 ± 9.66% b	HR (0-0-11-63-8)	22.81 ± 8.53% a	MR (0-3-66-13-0)
	Total	40.79 ± 11.55%	HR (0-1-29-139-44)	23.76 ± 8.57%	MR (0-3-172-36-2)

Note: I, Western hilly area; II, Central basin area; III, Central mountainous area; IV, Eastern inclined plain area. EHR, extremely high risk; HR, high risk; MR, medium risk; LR, low risk; HP, heavy pollution; MP, moderate pollution; SP, slight pollution; NP, non-polluting. The numbers behind the RSP evaluation level are the number of sample sites with no pollution–slight pollution–moderate pollution–severe pollution, and the numbers behind the RAC evaluation level are the number of samples with no risk–low risk–medium risk–high risk–extremely high risk. Different lowercase letters in the same column represent the results with statistical difference at *p* < 0.05.

## Data Availability

The original contributions presented in this study are included in the article/Supplementary Materials. Further inquiries can be directed to the corresponding authors.

## Supplementary Materials

The following supporting information can be downloaded at https://www.mdpi.com/article/10.3390/toxics13110969/s1, Figure S1: Proportion of soil at all sampling points with heavy metal pollution risks based on the RSP method; Figure S2: Proportion of heavy metals in different pollution risk levels (RSP); Figure S3: Proportion of soil at all sampling points with heavy metal pollution risks based on the RAC method; Figure S4: Proportion of heavy metals in different pollution risk levels (RAC); Table S1: Ecological risk assessment based on heavy metal speciation in soil.

## Abbreviations

The following abbreviations are used in this manuscript:

HMs	Heavy metals
Cd	Cadmium
Cu	Copper
Pb	Lead
Zn	Zinc
RSP	Secondary phase to primary phase ratio
RAC	Risk assessment code
EX	Exchangeable
CA	Carbonate-bound
OX	Fe-Mn oxide-bound
OR	Organic matter-bound
RE	Residual
CV	Coefficient of variation
