# Supplementary material for: Spatial Distribution Characteristics and Risk Assessment of Soil Heavy Metals from Long-Term Mining Activities: A Case Study of the Fengfeng Mining Area
Source: Toxics. 2025 Nov 10;13(11):969. doi: 10.3390/toxics13110969 (PMC12656562; doi:10.3390/toxics13110969)
Supplement: Supplementary file 1 [file toxics-13-00969-s001.zip › toxics-3920320-supplementary.pdf]

Supplementary material

Spatial Distribution Characteristics and Risk Assessment of Soil Heavy Metals from Long-Term Mining Activities: A Case Study of the Fengfeng Mining Area

Le Ren[1], Wenyu Qi[2*], Hongling Ye[1*]

[1] *School of life science and engineering, Handan University, Handan 056005, China*

[2] *Shandong Key Laboratory of Water Pollution Control and Resource Reuse, School of Environmental Science and Engineering, Shandong University, Qingdao 266237, China*

The following are included as supporting information for this paper:

number of pages: 5

number of figures: 4

number of tables: 1

* Corresponding author
E-mail address: wenyuqi@sdu.edu.cn (Wen-Yu Qi); yhl228007@hdc.edu.cn (Hong-Ling Ye)

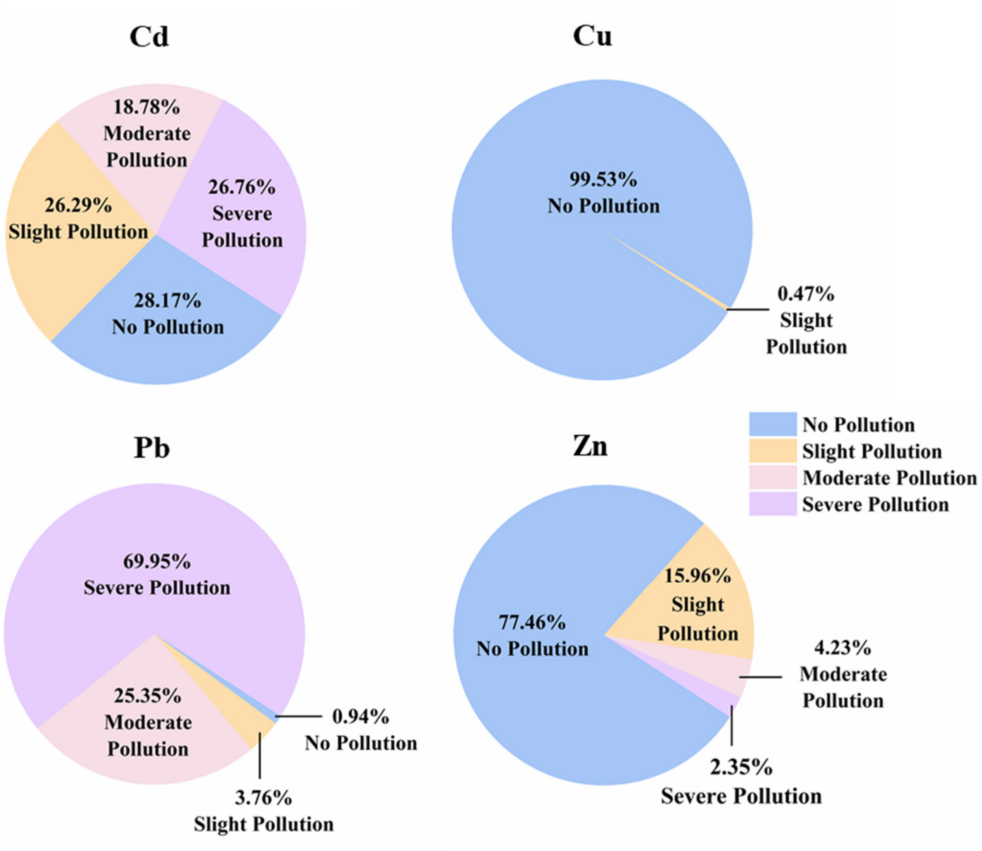

Figure S1 Proportion of soil at all sampling points with heavy metal pollution risks based on the RSP method

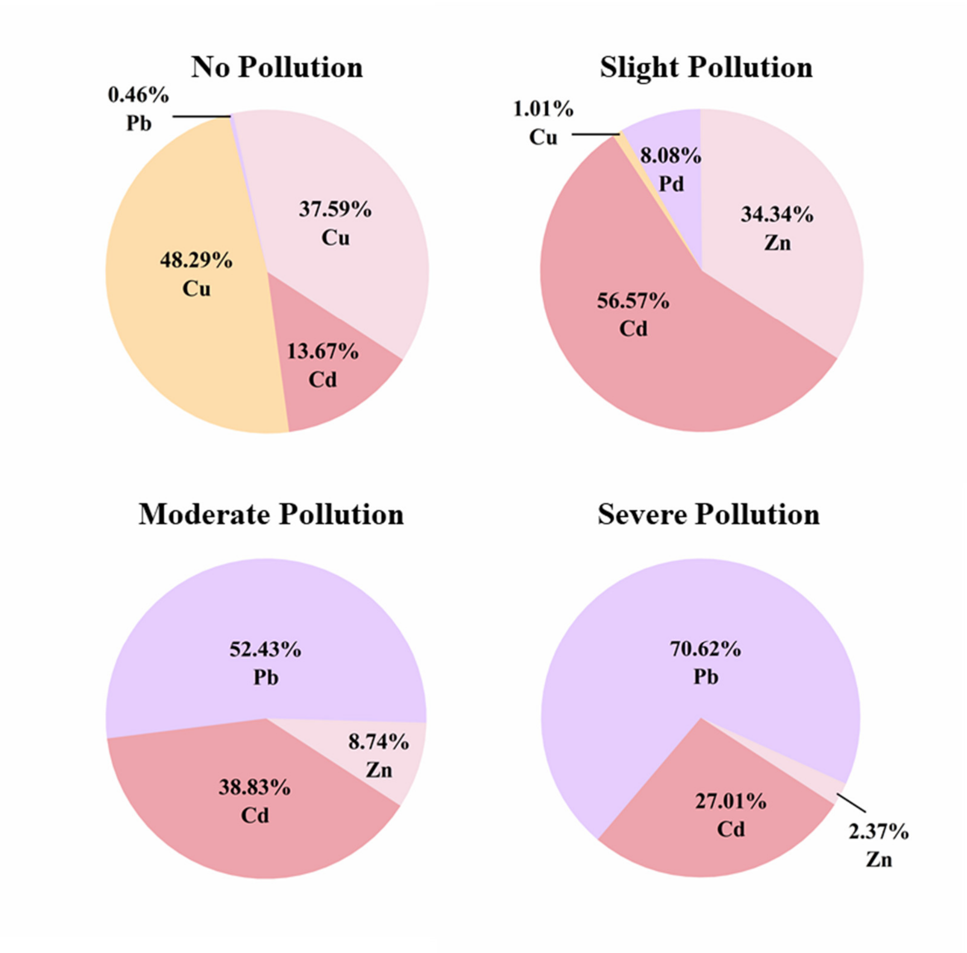

Figure S2 Proportion of heavy metals in different pollution risk levels (RSP)

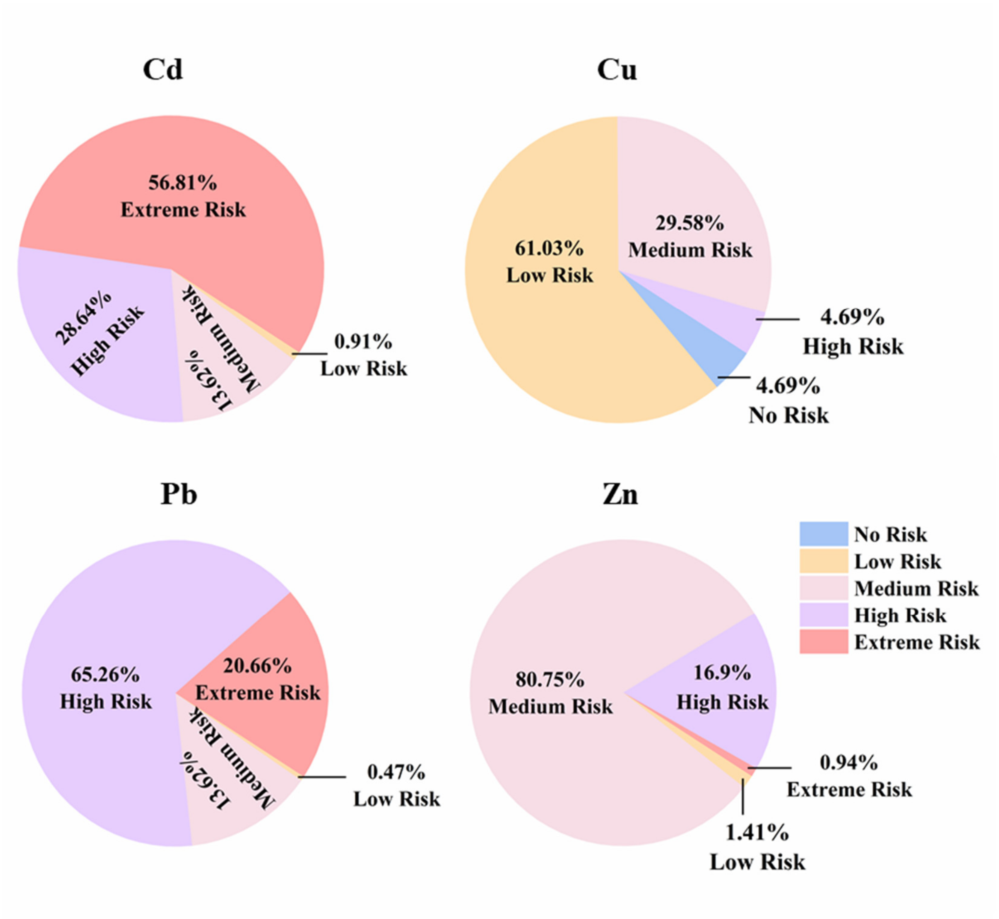

Figure S3 Proportion of soil at all sampling points with heavy metal pollution risks based on the RAC method

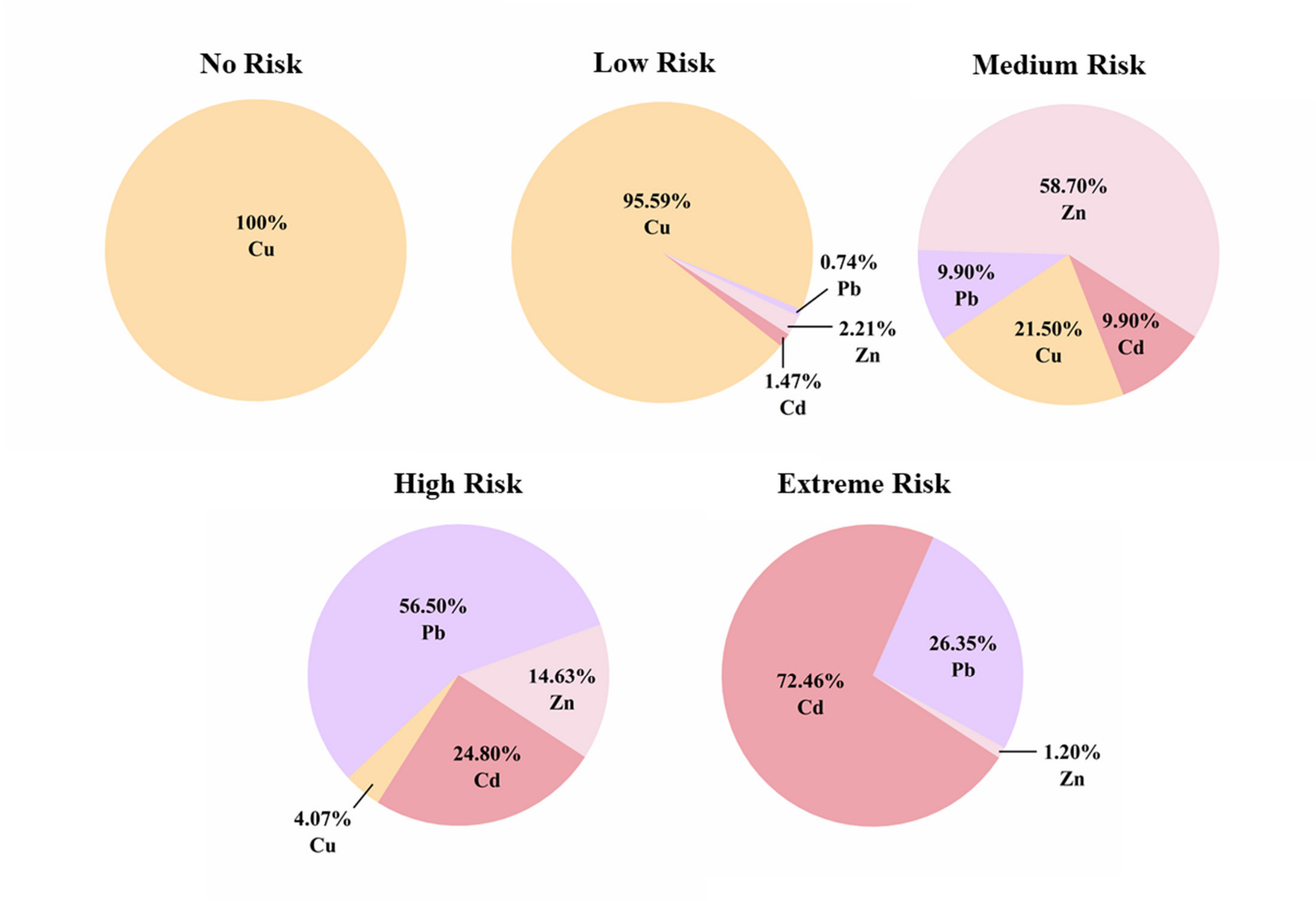

Figure S4 Proportion of heavy metals in different pollution risk levels (RAC)

Table S1. Ecological risk assessment based on heavy metal speciation in soil

HMs	Landform Subarea	HMs Speciations				
		EX	CA	OX	OR	RE
Cd	Ⅰ	0.080±0.048a	0.100±0.049b	0.027±0.021a	0.004±0.006a	0.137±0.117a
	Ⅱ	0.061±0.047ab	0.100±0.029b	0.035±0.046a	0.006±0.011a	0.147±0.167a
	Ⅲ	0.064±0.052ab	0.113±0.037ab	0.035±0.038a	0.004±0.003a	0.174±0.206a
	Ⅳ	0.050±0.035b	0.134±0.056a	0.030±0.010a	0.003±0.003a	0.214±0.264a
	Total	0.060±0.045	0.116±0.047	0.032±0.31	0.004±0.006	0.178±0.214
Cu	Ⅰ	1.187±2.368a	1.477±1.273b	0.496±0.599a	1.102±0.331a	24.03±13.91b
	Ⅱ	1.015±0.866a	2.276±2.425a	0.730±0.834a	1.418±0.599a	28.13±14.43ab
	Ⅲ	1.446±1.506a	1.321±1.550b	0.776±1.232a	1.215±0.548a	23.72±11.31b
	Ⅳ	1.219±1.697a	1.230±1.251b	0.818±0.525a	1.344±0.726a	34.13±15.88a
	Total	1.210±1.574	1.565±1.757	0.745±0.821	1.306±0.622	28.97±14.92
Pb	Ⅰ	7.720±4.930a	47.85±18.95a	23.25±8.258a	16.14±11.86a	13.10±8.937b
	Ⅱ	6.197±4.864ab	31.03±12.47b	18.31±12.16a	18.86±13.55a	18.18±10.38ab
	Ⅲ	5.164±2.598b	32.32±12.14b	22.75±16.75a	13.42±9.122a	21.91±19.91a
	Ⅳ	5.175±3.206b	31.68±9.505b	24.35±12.37a	18.56±11.53a	20.75±9.128a
	Total	5.761±3.918	33.62±13.38	22.22±13.16	17.21±11.82	19.37±12.79
Zn	Ⅰ	17.97±11.96b	21.05±2.785a	17.27±5.448ab	17.74±2.677a	102.1±24.61a
	Ⅱ	24.80±7.282a	18.49±8.540a	12.73±8.015b	18.38±2.701a	116.1±54.70a
	Ⅲ	25.48±9.449a	17.39±8.466a	20.40±10.40a	19.06±2.764a	92.96±41.87a
	Ⅳ	21.75±8.707ab	18.45±8.058a	14.36±8.716b	17.45±4.575a	113.0±46.76a
	Total	22.94±9.242	18.55±7.871	15.61±9.031	18.09±3.587	108.1±46.62

Note: I, Western hilly area; II, Central basin area; III, Central mountain area; Ⅳ, Eastern inclined plain area. EX extract exchangeable; CA, carbonate bound; OX, Fe-Mn oxide bound; OR, organic speciation; RE, residual speciation
